# Dental anxiety among children attending university-affiliated special needs and child dental clinics in Trinidad and Tobago: a cross-sectional study

**DOI:** 10.1186/s12903-025-06643-6

**Published:** 2025-07-26

**Authors:** Ramaa Balkaran, Satu Lahti, Visha Ramroop, Jorma I. Virtanen

**Affiliations:** 1 The University of the West Indies, St. Augustine, Trinidad and Tobago; 2 Department of Community Dentistry, Institute of Dentistry, University of Turku, Turku, Finland; 3 Faculty of Medicine, University of Bergen, Bergen, Norway

**Keywords:** Dental anxiety, Children with disabilities, Parents, Caregivers, Modified dental anxiety scale (MDAS)

## Abstract

**Background:**

The study investigated whether dental fear and anxiety (DFA) differed among children with and without disabilities in Trinidad and Tobago.

**Methods:**

For this cross-sectional study, the parents/caregivers of all 6–18-year-old children (*n* = 201) attending the Special Needs Dental Clinic and Child Dental Health Clinic were recruited. The Modified Dental Anxiety Scale (MDAS) was utilised on the accompanying adult. Multinomial regression models were used for analyses of MDAS sum score for three groups: low (5–9), moderate (10–18), and high anxiety (19–25). Disability was categorized as yes (any disability)/no. The covariables included Gender, Age of child, Ethnicity, accompanying adult (parent/caregiver), Reason for visit, Last visit, and Oral health rating of the child.

**Results:**

The parents/caregivers of children with disabilities (*n* = 101) and without (*n* = 100) responded. The mean age of the children with disabilities was 10.6 (3.4 SD) and 11.3 (2.8 SD) for the children without disabilities. Children with a disability were significantly more likely (OR: 3.7; CI: 1.9–7.5) to experience moderate level DFA than those without a disability. Also, children in the 6–12-year-old age group were more likely (OR: 5.6; CI: 1.1–27.1) to experience a high level of DFA than the 13–18-year-old children.

**Conclusions:**

Children with disabilities had a higher proportion of moderate levels of anxiety than those without. Consistent dental attendance at clinics using DFA techniques is suggested to reduce the development and persistence of DFA in this population.

## Background

Dental fear is an emotional response to a particular threat associated with dental treatment, whereas dental anxiety represents an excessive reaction to a perceived threat by patients undergoing dental care [1]. These terms are often used interchangeably as dental fear and anxiety (DFA) [1]. It is present in all age groups in the population and is a barrier to positive oral health outcomes [2]. DFA is a common problem in the child population experienced by approximately one-third of young children globally [3]. The global prevalence of pooled DFA is 23.9% and DFA has been shown to affect preschool and school children more than adolescents [4]. A study in Trinidad and Tobago, reported similar levels i.e., just over one-third of the children aged 10–16 years old had high levels of DFA [5]. Globally, the prevalence of DFA in adults was estimated at 15.3% [6]. DFA is challenging for dentists providing treatment and can compromise the oral health in uncooperative children [7].

Also, a patient with high DFA may expect more pain or experience greater pain during treatment and post-treatment pain [8]. Further, a history of unpleasant dental experiences, such as pain and high dental caries experience, has been shown to have an important role in both the avoidance of dental treatment and the development of dental anxiety [9]. The avoidance of dental treatment has also been associated with DFA in the adult population, and a decrease in DFA resulted in more regular dental attendance [10].

If a mother’s DFA is high, it can influence her child’s coping ability in the dental setting [7, 11]. Parents and caregivers are often central to accessing dental care for children with and without disabilities. Children who are anxious about dental procedures may be less likely to seek and receive dental care. This delayed attendance can worsen the oral health and overall well-being, leading to a ‘vicious cycle’ of dental fear associated with dental visits [12, 13]. This vicious cycle may also lead to poorer self-rated oral health [14].

DFA is common in children and adolescents with disabilities [15–17]. Research indicates that over two-thirds of children with autism are estimated to experience clinically significant DFA [17], and about one-fifth of those with intellectual disabilities demonstrate DFA [16]. Research has shown that the levels of anxiety in caregivers of children with and without disabilities were similar, and those children whose caregivers have higher trait anxiety levels reported higher anxiety levels [18]. There is a general paucity of research on DFA in children with disabilities, and no available data is currently available on this population in Trinidad.

The study aimed to investigate the dental fear and anxiety among children aged 6–18 years old attending the Special Needs Dental Clinic and the Child Dental health Clinic at the University of the West Indies (UWI) School of Dentistry.

## Methods

This was a cross-sectional survey conducted between July 2022 – February 2023. The sample was obtained from all consecutive parents/caregivers of children 6–18 years old attending the University of the West Indies (UWI) School of Dentistry Special Needs Dental Clinic and the Child Dental Health Clinic. The questionnaires measured dental fear and anxiety and variables such as demographics (gender, age, ethnicity), accompanying adult (parent/caregiver), reason for visit, dental attendance (last visit to the dentist), and rating of child’s oral health. The questionnaires were administered to the parents/caregivers (*n* = 201) by two clinicians face-to-face in the clinics, and were completed independently by the participants. The PI was trained in body language and voice control, including intonation and neutral expressions, and the other clinician was also trained to ensure that parents’ responses were uninfluenced by their involvement in the data collection. Face-to-face administration allowed for the clarification of any questions that parents or caregivers may have had in real-time, ensuring that participants fully understood the questions being asked. Accompanying adults and their children under 6 or over 18 years old were excluded from the study. The accompanying adults of the patients responded to the questionnaires as in the study of Balkaran et al. [19]. All respondents in the accompanying adult group responded.

### Outcome variables

The DFA was measured using the valid and reliable Modified Dental Anxiety Scale (MDAS) [20, 21]. The questions in the MDAS are as follows:

- Item 1: if your child went to their dentist for treatment tomorrow, how would they feel?
- Item 2: if your child were sitting in the waiting room (waiting for treatment), how would they feel?
- Item 3: if your child were about to have a tooth drilled, how would they feel?
- Item 4: if your child were about to have their teeth scaled and polished, how would they feel?
- Item 5: if your child were about to have a local anaesthetic injection in their gum, above an upper back tooth, how would they feel?

The MDAS is a frequently employed psychometric instrument for assessing the dental anxiety levels of the general population, with multiple international studies that can be used for comparison [21]. It is also widely used, and it is possible to compare the scales of DFA with conversion Tables [22, 23]. Further, the MDAS as a proxy has been recently used [24]. The MDAS used a 5-point Likert scale which ranged from ‘not anxious’ to ‘extremely anxious’. The responses were coded with the numbers: 1 (= not anxious), 2 (= slightly anxious), 3 (= fairly anxious), and 4 (= very anxious) 5 (= extremely anxious). The total scores ranged from 5 to 25. The variable was trichotomized as follows: a score of less than 10 was considered ‘no to low dental anxiety’, whereas those lying between 11 and 18 represented ‘moderate dental anxiety’, similar to previous studies [23, 25, 26]. Scores of 19 and greater represented ‘high anxiety’ [27]. The two separate factors of the MDAS: the anticipatory dental anxiety (items 1 and 2; score range = 2–10) and treatment dental anxiety (items 3, 4, and 5; score range = 3–15) were also calculated as sums of the item responses [26, 28].

### Other variables

The analyses considered the following variables about the children: disability, gender, age, ethnicity, accompanying adult (parent/caregiver), reason for visit, last visit to the dentist, and rating of the child’s oral health.

### Statistical analysis

The distribution of the participants according to outcome and covariables, was presented using frequencies and percentages (Table 1). The means, standard deviations, medians, and the first and third quartiles of MDAS total sum and treatment-related and anticipatory sum scores were calculated. Then, a multinomial regression model was fitted for MDAS for the three groups: 5–9 (low), 10–18 (moderate), and 19+ (high anxiety), where the reference category was low anxiety. Disability (yes = 1) was the factor. The covariables, dichotomized as 0 and 1 were: gender (male = 1), (female = 0), Age (6–12 = 1), (13–18 = 0), Ethnicity (Afro-Caribbean = 1) (Indo-Caribbean + Mixed = 0), Accompanying adult caregiver (Mother = 1) (Father + Caregiver = 0), Reason for visit (Pain + Filling + Cleaning + Other = 1) (Check-up = 0), Last visit to the dentist (Less than one year + One year + Two years + Pain/ Emergency = 1) (Never = 0), Rating of child’s oral health (Excellent + Very Good + Good = 1) ( Fair + Poor = 0). Data were analysed using SPSS version 26.0 (IBM SPSS Statistics for Windows, Armonk, NY: IBM Corp.). The statistical significance level of the study was set at 0.05.

### Ethical considerations

Written informed consent was obtained from the respondents who fit the inclusion criteria. No data linked participants’ identities to the questionnaires. The study followed the principles of the Helsinki Declaration. The protocol for the study was approved by the University of the West Indies Ethics Committee (CREC-SA.1609/05/2022).

## Results

There were 101 responses collected on children with disabilities and 100 without disabilities; all the invited parents/caregivers agreed to participate in this study. Autism was the most commonly reported disability (21.9%), and 22.9% were non-verbal among the children with disabilities. Other disabilities included were ADHD (8.0%), Global developmental delay (7.5%), Down Syndrome (6.5%), Intellectual disabilities (6.5%), speech delay (4%), Cerebral Palsy (2.5%). Remaining disabilities occurred less frequently (≤ 1%).

The mean age was 10.6 (3.4 SD) among children with disabilities, whereas among those without disabilities, the mean age was 11.3 (2.8 SD). The majority of children (68.3%) with disabilities were male, while just over half of children (52.0%) without disabilities were female. Of all children, just under one-third had never visited the dentist before this visit, according to their parents/ caregivers. Those without a disability comprised the majority (44.0%) of first-time attendees, while the corresponding percentage for those with a disability was 20.8%. The mean MDAS score for the total population was 11.1 (4.8 SD), and most (54.7%) had low anxiety. The mean MDAS score was higher in the children with disabilities (12.0 ± 4.6) who attended for a “check-up” than those without disabilities (9.0 ± 4.3). The proportion of children experiencing moderate anxiety was higher in those with disabilities than in those without disabilities (Table 1).

**Table 1:** Frequency (N, %), MDAS total score mean (SD), percentage low, moderate, and high DFA (*n* = 201)

Variables	*N*	%	Mean	SD	Low anxiety %	Moderate anxiety %	High anxiety %
**Disability** Yes	101	50.2	12.2	4.6	18.4	27.4	4.5
No	100	49.8	9.9	4.7	30.3	15.9	3.5
**Gender** Male	117	58.2	11.3	5.0	27.9	24.4	6.0
Female	84	41.8	10.8	4.5	20.9	18.9	2.0
**Age** 6–12-year-olds	132	65.7	11.9	4.9	26.9	31.8	7.0
13-18-year-olds	69	34.3	9.5	4.1	21.9	11.4	1.0
**Ethnicity** Afro-Caribbean	69	34.3	10.8	4.4	16.9	15.4	2.0
Indo-Caribbean	46	22.9	10.8	5.3	11.4	9.5	2.0
Mixed	86	42.8	11.4	4.8	20.4	18.4	4.0
**Parent/caregiver** Mother	137	68.2	11.6	5.0	30.8	30.3	7.0
Father	34	16.9	9.8	3.9	9.5	7.5	0.0
Caregiver	30	14.9	9.8	3.9	8.5	5.5	1.0
**Reason for visit** Pain, Filling, Cleaning, Other	87	43.3	10.8	4.9	21.9	17.9	3.5
Check-up	114	56.7	11.3	4.7	26.9	25.4	4.5
**Last visit to the dentist** Less than one year, One year, Two years, Pain/ Emergency	136	67.7	10.9	4.8	34.3	28.9	4.5
Never	65	32.3	11.3	4.7	14.4	14.4	3.5
**Rating of child’s oral health** Excellent, Very Good, Good	101	50.2	10.5	4.4	25.4	22.4	2.5
Fair, Poor	100	49.7	11.6	5.1	23.4	20.9	5.5

Reason for visit: Pain + Filling + Cleaning + Other have been combined (Check-up has not been changed), Last visit to the dentist: Less than one year + One year + Two years + Pain/Emergency have been combined (Never has not been changed), Rating of child’s oral health: Excellent + Very Good + Good have been combined and then Fair + Poor have been combined

The responses from the parents/caregivers suggested that children with disabilities had higher mean and median dental anxiety levels than those without disabilities in the total as well as in anticipatory and treatment-related dental anxiety. The children with disabilities had over two points higher mean MDAS scores than children without disabilities (Table 2).

**Table 2:** MDAS total (5–25), treatment-related (3–15), and anticipatory (2–10) factor scores, means (SD), and medians (*n* = 201)

Variables	Children with disabilities *n* = 101 (Valid %)		Children without disabilities *n* = 100 (Valid %)				*p*-value *n* = 201	
	Mean (SD)	Md (Q1-Q3)	Mean (SD)		Md (Q1-Q3)			
**MDAS total sum**	12.2 (4.6)	11 (9–15)	9.9 (4.7)		8.5 (6–13)			**0.000**
**MDAS: treatment**	8.5 (3.3)	8 (6–11)	6.8 (3.2)		6.0 (4–9)			**0.027**
**MDAS: anticipatory**	3.8 (2.1)	3 (2–5)	3.2 (1.8)		2.0 (2–4)			**0.000**

*p*-value < 0.05 for the Kruskal-Wallis tests between children with and without disabilities among children visiting the University of the West Indies (UWI) Special Needs Clinic and Child Dental Health Clinic in Trinidad

Children with disabilities were almost four times more likely (OR: 3.7; CI: 1.9–7.5) to experience DFA at a moderate level than children without disabilities (Table 3).

**Table 3:** Multinomial analysis of parents/caregivers with low anxiety (MDAS score 5–9) as the reference category (*n* = 201)

	Variables	B	S.E.	Wald	df	OR	95% CI	*p*-value
**Moderate anxiety**	**Intercept**	-1.1	0.6	4.2	1			**0.045**
	**Gender**	-0.3	0.3	1.0	1	0.7	0.4–1.4	0.328
	**Age**	0.7	0.3	4.8	1	2.1	1.1-4.0	**0.029**
	**Disability**	1.3	0.4	13.6	1	3.7	1.9–7.5	**0.000**
	**Ethnicity**	-0.1	0.3	0.2	1	0.9	0.5–1.7	0.676
	**Parent/caregiver**	0.5	0.3	1.9	1	1.6	0.8–3.2	0.163
	**Reason for visit**	0.2	0.3	0.4	1	1.2	0.6–2.4	0.520
	**Last visit to the dentist**	-0.5	0.4	2.1	1	0.6	0.3–1.2	0.152
	**Rating of child’s oral health**	0.1	0.3	0.1	1	1.1	0.6–2.1	0.759
**High anxiety**	**Intercept**	-4.4	1.3	11.5	1			**0.001**
	**Gender**	0.7	0.7	1.1	1	2.0	0.6–7.3	0.284
	**Age**	1.7	0.8	4.5	1	5.6	1.1–27.1	**0.033**
	**Disability**	1.1	0.7	2.6	1	3.0	0.8–11.8	0.106
	**Ethnicity**	-0.7	0.7	1.2	1	0.5	0.1–1.8	0.272
	**Parent/caregiver**	1.5	0.8	3.5	1	4.6	0.9–23.0	0.062
	**Reason for visit**	0.4	0.6	0.5	1	1.5	0.5–4.8	0.481
	**Last visit to the dentist**	-0.8	0.7	1.5	1	0.4	0.1–1.6	0.225
	**Rating of child’s oral health**	-0.7	0.6	1.4	1	0.5	0.1–1.6	0.232

Anxiety range (5–9 (low), 10–18 (moderate), and 19+ (high anxiety) was the dependent variable (the reference category is low anxiety). Disability (yes = 1 was the factor) The covariables were dichotomized as 0 and 1 were: gender (male = 1) Age (6–12 = 1), Ethnicity (Afro-Caribbean = 1), Parent/caregiver (Mother = 1), Reason for visit (Pain + Filling + Cleaning + Other = 1), Last visit to the dentist (Two years/ less = Less than one year + One year + Two years + Pain/ Emergency = 1), Rating of child’s oral health (Excellent + Very Good + Good = 1). Abbreviations: SE, standard error; df, degree of freedom; OR, odds ratio, CI, confidence interval

Additionally, those who were younger were almost six times more likely (OR: 5.6; CI: 1.1–27.1) to experience a high level of DFA than the older age group of 13–18-year-olds. The younger age group was twice more likely than the older age group to have moderate level DFA.

## Discussion

The main finding of this cross-sectional study showed that children with a disability were almost four times more likely to experience a moderate level DFA than those without a disability in Trinidad. Additionally, younger children, in the 6–12-year-old age group were more likely to experience both a moderate and high level DFA than the older age group of 13–18-year-olds.

Several instruments and study designs have been used to assess DFA in children and adolescent groups globally [4]. Generally, the reported prevalence of DFA is not significantly impacted by the chosen assessment tool [3]. A previous study conducted in the Child Dental Health Clinic at the University of the West Indies reported similar levels of anxiety in children aged 10–16 years old [5]. A longitudinal study among 7–9-year-old Swedish children found the prevalence of DFA was 7% at age seven and 8% at age nine with the parental version of the CFSS-DS [29]. Our study showed a higher prevalence of moderate anxiety than an Italian study using Corah’s Dental Anxiety Scale [16]. The epidemiological study conducted by the researchers indicated that the increase in dental anxiety correlated directly with elevated levels of intellectual disabilities. In contrast, a more pronounced decrease in dental anxiety was observed among older children (ages 10–20) compared to the younger cohort (ages 0–10) [16]. It should, however, be noted that the variation in prevalence and mean scores of DFA may be based on the use of different scales and their threshold for DFA, particularly the determined cut-off points [1], as well as the age of the study participants [16].

### Children from the special needs clinic

Further, the accompanying parents/caregivers reported that children with disabilities had higher anxiety levels than those without disabilities in both anticipatory and treatment-related dental anxiety. This was comparable to Rantavuori et al. [30] who found that the injection, local anaesthetic, and drill were the most anxiety-provoking, however in their study general dental fear was less prevalent in the adolescent population. The responses from the parents/caregivers in this study suggested that children with disabilities had higher anxiety levels than those without, which is consistent with recent research [31]. Autism was the most commonly reported disability (21.9%) in this study. Given that significant dental anxiety has been associated in 7–13-year-olds with autism and co-occurring anxiety disorders, the proportion of anxiety in the children from the special needs dental clinic is reasonably higher than those in the paediatric clinic [17]. Generally, younger children have higher values of DFA [1]. This was the case in our study: children in the age group of 6–12 years old experienced significantly more moderate and high anxiety than the older children (13-18-year-olds). Additionally, in our study, there was no significant difference in the DFA between males and females, consistent with other research [3].

Children with disabilities may not be capable of comprehending dental treatment or may have oral aversions due to their orofacial conditions which can reduce cooperation with the dentist [31]. Our study showed that the proportion of moderate-level DFA was more common in children with a disability than those without. This may have been due to the fact that children with disabilities may have previously been hospitalized or had medical experience, both of which may contribute to DFA in a clinical environment [32].

### Children from the child dental health clinic

The main difference between the Child Dental Health Clinic and Special Needs Dental Clinic patient populations was their dental attendance, as many children at the Child Dental Health Clinic were first-time attendees. The Child Dental Health Clinic has a daily clinic and emergency services on various days with routine, and orthodontic sessions. However, the Special Needs Dental Clinic delivers routine and emergency treatment only three days per week. During this research, a grant from a non-governmental Organisation (NGO), Community Chest Limited, covered all treatment costs for children at the Special Needs Dental Clinic, but there was a cost for children at the Child Dental Health Clinic. This difference in dental attendance could have been due to cost. Additionally, research has shown that mothers of children may underestimate the prevalence of their children’s DFA, and this could have impacted the lower prevalence of DFA [33].

Patterns of symptomatic use of dental services have been associated with continued avoidance of dental management, which can lead to the persistence or worsening of dental fear [33]. Although children without disabilities who have had prior dental visits may develop DFA, the symptomatic treatment will be traumatic due to negative family influences [34]; this was not observed in children without disabilities. Concerning gender, most children from the Child Dental Health Clinic were female. While some studies have found females to have a higher DFA [1], this was not seen in our results, as gender differences have not been consistent in previous studies [4, 35].

### Dental utilisation in Trinidad and Tobago

There is a high demand and utilization of services provided at the UWI dental clinics for children, possibly owing to the generally high costs of private dental care and unpredictable services in government clinics. Dental care in Trinidad and Tobago is available through private clinics, government health centres, and the UWI dental school. Dental treatment for children from two to age 12 includes preventive, restorative, and extraction services in government clinics, delivered by dental nurses [36]. Children over 12 are treated by dentists, and the care is often limited to extractions with long wait times and restricted service days. Cost is a barrier to accessing dental treatment and may have accounted for the difference in the frequency of dental visits in both clinics. In 2021, patients with disabilities received free treatment at the UWI Special Needs Dental Clinic. We did not assess the service utilisation of the children with disabilities before 2021, when there was a cost at the UWI Special Needs Dental Clinic. The higher levels of DFA in their group may have arisen from previous delayed attendance. Further, this increased DFA may have arisen from the need for more complex treatments owing to delay, since untreated dental issues can worsen over time, particularly in children.

Some patients are uncooperative owing to their DFA and require treatment under sedation or general anaesthesia when other management techniques have failed. However, this is not always possible in a developing country like Trinidad and Tobago [31]. The waiting list for oral surgery under sedation or general anaesthesia in Trinidad can vary between two to three years due to limited resources, and only non-pharmacologic behaviour guidance techniques are employed. Culturally, parents often influence their children to perceive dental visits negatively, sometimes using the notion of going to the dentist to “pull out a tooth” as a threat [5]. Typically, this is a common practice in Trinidad. This is problematic, especially since it may lead to the development of DFA through this negative information [7].

### Strengths and Limitations

There is limited research on the DFA on children with disabilities, and this study provides new information on this vulnerable population of children in Trinidad and Tobago. Although caregivers of PWDs may overestimate their DFA, paired caregiver-patient scores have not revealed significant differences [37], suggesting that caregivers can accurately gauge their child’s fear. We used experienced and calibrated examiners to administer the questionnaires using a valid and reliable methodology with similar research. A strength of the multinomial analysis was the combination of the groups compared to the dichotomy. The results from this study cannot be generalized since the results may have been different if both clinics offered treatment at no cost. We recommend longitudinal studies for future research to compare children with and without disabilities. Furthermore, there is a need to transition from paediatric to adult care when children with disabilities attain that age, to ensure continuity of care and reduce neglect in this vulnerable population [38].

### Recommendations

Regular dental care is suggested to minimise the development of DFA in childhood or adolescence [39]. This underscores the value of having a dedicated special needs clinic for undergraduate training to improve the access to delivery of dental care in this population and regular attendance. Globally, the delivery of special needs dentistry varies greatly; a recent review showed that the distribution of provision of oral care mainly occurred in developed countries such as the USA, Canada, Australia, and some European countries, which delivered some component of special needs dentistry but were unable to determine the extent to which this was delivered [40]. The UWI Special Needs Dental Clinic endeavours to provide dental treatment as comfortably as possible to reduce the development of dental anxiety.

## Conclusions

Children with disabilities experienced significantly higher levels of moderate levels of anxiety than those without in Trinidad and Tobago. Consistent dental attendance at clinics using DFA-minimizing methods is suggested to reduce the development and persistence of DFA in this population. Healthcare providers should assess and address patients’ DFA. Evaluating DFA enhances care and reduces barriers stemming from dental fear misperceptions.

## Data Availability

The datasets used and/or analyzed during the current study are available from the corresponding author on reasonable request.

## Abbreviations

CI: Confidence Interval
DFA: Dental Fear and Anxiety
MDAS: Modified Dental Anxiety Scale
NGO: Non-Governmental Organisation
OHRQoL: Oral Health-Related Quality of Life
OR: Odds Ratio
SD: Standard deviation
UWI: The University of the West Indies

## References

[1] Cianetti S, Lombardo G, Lupatelli E, Pagano S, Abraha I, Montedori A, Caruso S, Gatto R, De Giorgio S, Salvato R. Dental fear/anxiety among children and adolescents. A systematic review. Eur J Paediatr Dent. 2017;18(2):121–30. 10.23804/ejpd.2017.18.02.07. 28598183 10.23804/ejpd.2017.18.02.07

[2] Stein Duker LI, Grager M, Giffin W, Hikita N, Polido JC. The relationship between dental fear and anxiety, general anxiety/fear, sensory Over-Responsivity, and oral health behaviors and outcomes: A conceptual model. Int J Environ Res Public Health. 2022;18(4):19. 10.3390/ijerph19042380. 10.3390/ijerph19042380 PMC8872083 35206566

[3] Sun IG, Chu CH, Lo ECM, Duangthip D. Global prevalence of early childhood dental fear and anxiety: A systematic review and meta-analysis. J Dent. 2024;142:104841. 10.1016/j.jdent.2024.104841. 38246307 10.1016/j.jdent.2024.104841

[4] Grisolia BM, Dos Santos APP, Dhyppolito IM, Buchanan H, Hill K, Oliveira BH. Prevalence of dental anxiety in children and adolescents globally: A systematic review with meta-analyses. Int J Paediatr Dent. 2021;31(2):168–83. 10.1111/ipd.12712. 33245591 10.1111/ipd.12712

[5] Kowlessar A, Roopnarine G, Ramroop V, Balkaran R, Harracksingh A, Naidu R. Dental anxiety/fear among children aged 10 to 16 in a dental hospital in Trinidad. CMJ. 2013;75(1):1–5.

[6] Silveira ER, Cademartori MG, Schuch HS, Armfield JA, Demarco FF. Estimated prevalence of dental fear in adults: A systematic review and meta-analysis. J Dent. 2021;108:103632. 10.1016/j.jdent.2021.103632. 33711405 10.1016/j.jdent.2021.103632

[7] Themessl-Huber M, Freeman R, Humphris G, MacGillivray S, Terzi N. Empirical evidence of the relationship between parental and child dental fear: a structured review and meta-analysis. Int J Paediatr Dent. 2010;20(2):83–101. 10.1111/j.1365-263X.2009.00998.x. 20384823 10.1111/j.1365-263X.2009.00998.x

[8] Lin CS, Wu SY, Yi CA. Association between anxiety and pain in dental treatment: A systematic review and Meta-analysis. J Dent Res. 2017;96(2):153–62. 10.1177/0022034516678168. 28106507 10.1177/0022034516678168

[9] Skaret E, Raadal M, Berg E, Kvale G. Dental anxiety and dental avoidance among 12- to 18-year-olds in Norway. Eur J Oral Sci. 1999;107(6):422–8. 10.1046/j.0909-8836. 1999.eos107602. x. 10625100 10.1046/j.0909-8836.1999.eos107602.x

[10] Liinavuori A, Tolvanen M, Pohjola V, Lahti S. Changes in dental fear among Finnish adults: a National survey. Community Dent Oral Epidemiol. 2016;44(2):128–34. 10.1111/cdoe.12196. 26482701 10.1111/cdoe.12196

[11] Besiroglu-Turgut E, Kayaalti-Yuksek S, Bulut M. Evaluation of the relationship between dental anxiety and oral health status of mothers and their children. BMC Oral Health. 2024;24:749. 10.1186/s12903-024-04530-0. 38943136 10.1186/s12903-024-04530-0 PMC11212381

[12] Berggren U, Carlsson SG. Psychometric measures of dental fear. Community Dent Oral Epidemiol. 1984;12:319–24. 6593151 10.1111/j.1600-0528.1984.tb01463.x

[13] Berggren U, Carlsson SG. Usefulness of two psychometric scales in Swedish patients with severe dental fear. Community Dent Oral Epidemiol. 1985;13(2):70–4. 3857151 10.1111/j.1600-0528.1985.tb01679.x

[14] Armfield JM, Stewart JF, Spencer AJ. The vicious cycle of dental fear: exploring the interplay between oral health, service utilization and dental fear. BMC Oral Health. 2007;7:1. 10.1186/1472-6831-7-1. 17222356 10.1186/1472-6831-7-1 PMC1784087

[15] Corcoran M, Karki S, Harila V, Luoto A, Ylikontiola L, Sándor GK, et al. Dental fear among adolescents with cleft. Int J Paediatr Dent. 2021;31(6):716–23. 10.1111/ipd.12782. 33730383 10.1111/ipd.12782

[16] Fallea A, Zuccarello R, Calì F. Dental anxiety in patients with borderline intellectual functioning and patients with intellectual disabilities. BMC Oral Health. 2016;16(1):114. 10.1186/s12903-016-0312-y. 27809836 10.1186/s12903-016-0312-y PMC5093997

[17] Park Y, Guzick AG, Schneider SC, Fuselier M, Wood JJ, Kerns CM, et al. Dental anxiety in children with autism spectrum disorder: Understanding frequency and associated variables. Front Psychiatry. 2022;13. 10.3389/fpsyt.2022.838557. 10.3389/fpsyt.2022.838557 PMC9021783 35463526

[18] Pinho RCM, da Silva Barbosa AC, Caldas-Júnior AF, Vasconcelos MMVB, Cimões R, Santos MTBRD. State, trait, and dental anxiety in caregivers of individuals with disabilities. Spec Care Dentist. 2017;37(4):168–75. 10.1111/scd.12230. 28636131 10.1111/scd.12230

[19] Balkaran R, Lahti S, Ramroop V, Virtanen JI. Oral health-related quality of life in children attending university special needs and paediatric dental clinics in Trinidad and tobago: A parental perspective. Acta Odontol Scand. 2025;84:1–8. 10.2340/aos.v84.43009. 40014424 10.2340/aos.v84.43009 PMC11926418

[20] Humphris GM, Morrison T, Lindsay SJE, Sep. 12(3): 143–50. 7584581

[21] Humphris GM, Freeman R, Campbell J, Tuutti H, D’Souza V. Further evidence for the reliability and validity of the modified dental anxiety scale. Int Dent J. 2000;50(6):367–70. 10.1111/j.1875-595x.2000.tb00570.x. 11197195 10.1111/j.1875-595x.2000.tb00570.x

[22] Freeman R, Clarke HM, Humphris GM. Conversion tables for the Corah and modified dental anxiety scales. Community Dent Health. 2007;24(1):49–54. 17405471

[23] Seppänen S, Vuorenmaa K, Suominen A, Ogawa M, Pohjola V, Rantavuori K, Karlsson H, Karlsson L, Satu Lahti. Concordance of fathers and mothers in the assessment of their 5-Year-Old child’s dental fear. Dent J. 2024;12(3):53. 10.3390/dj12030053. 10.3390/dj12030053 PMC10968815 38534277

[24] AlAzmah A, Sharanesha RB, Abushanan A, Khojah Ab D, Aa, Almakenzi AA, Alqarni AS, Alagla M, Al Ghwainem A, Alghamdi S. Comparison of parental and children’s dental anxiety levels using the modified dental anxiety scale and modified short State-Trait anxiety inventory (EMOJI) scale. Children. 2024;11(12):1532. 10.3390/children11121532. 39767961 10.3390/children11121532 PMC11727085

[25] Chowdhury CR, Khijmatgar S, Chowdhury A, Harding S, Lynch E, Gootveld M. Dental anxiety in first- and final-year Indian dental students. BDJ Open. 2019;5:15. 10.1038/s41405-019-0017-9. 31632694 10.1038/s41405-019-0017-9 PMC6795851

[26] Kankaanpää R, Auvinen J, Rantavuori K, Jokelainen J, Karppinen J, Lahti S. Pressure pain sensitivity is associated with dental fear in adults in middle age: findings from the Northern Finland 1966 birth cohort study. Community Dent Oral Epidemiol. 2019;47(3):193–200. 10.1111/cdoe.12443. 30549076 10.1111/cdoe.12443

[27] King K, Humphris GM. Evidence to confirm the cut-off for screening dental phobia using the modified dental anxiety scale. Soc Sci Dentistry. 2010;1:21–8.

[28] Lahti SM, Tolvanen MM, Humphris G, Freeman R, Rantavuori K, Karlsson L, Karlsson H. Association of depression and anxiety with different aspects of dental anxiety in pregnant mothers and their partners. Community Dent Oral Epidemiol. 2020;48(2):137–42. 10.1111/cdoe.12511. 31809556 10.1111/cdoe.12511

[29] Dahlander A, Soares F, Grindefjord M, Dahllöf G. Factors associated with dental fear and anxiety in children aged 7 to 9 years. Dent J (Basel). 2019;7(3):68. 10.3390/dj7030068. 31266156 10.3390/dj7030068 PMC6784363

[30] Rantavuori K, Lahti S, Hausen H, Seppä L, Kärkkäinen S. Dental fear and oral health and family characteristics of Finnish children. Acta Odontol Scand. 2004;62(4):207–13. 10.1080/00016350410001586. 15513417 10.1080/00016350410001586

[31] American Academy of Pediatric Dentistry. Management of dental patients with special health care needs. The reference manual of pediatric dentistry. Chicago, Ill.: AAPD; 2024. pp. 343–50.

[32] Shindova MP, Belcheva AB. Dental fear and anxiety in children: a review of the environmental factors. Folia Med (Plovdiv). 2021;63(2):177–82. 10.3897/folmed.63. e54763. 33932006 10.3897/folmed.63.e54763

[33] Fu SW, Li S, Shi ZY, He QL. Interrater agreement between children’s self-reported and their mothers’ proxy-reported dental anxiety: a Chinese cross-sectional study. BMC Oral Health. 2023;23(1):139. 10.1186/s12903-023-02834-1. 36899301 10.1186/s12903-023-02834-1 PMC10007847

[34] Poulton R, Waldie KE, Thomson WM, Locker D. Determinants of early- vs late-onset dental fear in a longitudinal-epidemiological study. Behav Res Ther. 2001;39:777–85. 11419609 10.1016/s0005-7967(00)00060-7

[35] Klingberg G, Broberg AG. Dental fear/anxiety and dental behaviour management problems in children and adolescents: a review of prevalence and concomitant psychological factors. Int J Paediatr Dent. 2007;17(6):391–406. 10.1111/j.1365-263X.2007.00872.x. 17935593 10.1111/j.1365-263X.2007.00872.x

[36] Government of the Republic of Trinidad and Tobago, Ministry of Health. Dental Services. [Cited 10 Nov 2024] Available from: https://health.gov.tt/services/dental-services

[37] Martin MD, Kinoshita-Byrne J, Getz T. Dental fear in a special needs clinic population of persons with disabilities. Spec Care Dentist. 2002 May-Jun;22(3):99–102. 10.1111/j.1754-4505.2002.tb01170.x. 10.1111/j.1754-4505.2002.tb01170.x 12240894

[38] Borromeo GL, Bramante G, Betar D, Bhikha C, Cai YY, Cajili C. Transitioning of special needs paediatric patients to adult special needs dental services. Aust Dent J. 2014;59(3):360–5. 10.1111/adj.12197. 24889651 10.1111/adj.12197

[39] Seligman LD, Hovey JD, Chacon K, Ollendick TH. Dental anxiety: an understudied problem in youth. Clin Psychol Rev. 2017;55:25–40. 10.1016/j.cpr.2017.04.004. 28478271 10.1016/j.cpr.2017.04.004

[40] Scepanovic T, Mati S, Ming ALC, Yeo PYS, Nguyen D, Aria M, et al. The global distribution of special needs dentistry across dental school curricula. Spec Care Dentist. 2024 Jul-Aug;44(4):1191–210. 10.1111/scd.12973. 10.1111/scd.12973 38385902

