# Correction: Dental anxiety among children attending university-affiliated special needs and child dental clinics in Trinidad and Tobago: a cross-sectional study

**DOI:** 10.1186/s12903-025-06746-0

**Published:** 2025-08-15

**Authors:** Ramaa Balkaran, Satu Lahti, Visha Ramroop, Jorma I. Virtanen

**Affiliations:** 1 The University of the West Indies, St. Augustine, Trinidad and Tobago; 2 Department of Community Dentistry, Institute of Dentistry, University of Turku, Turku, Finland; 3 Faculty of Medicine, University of Bergen, Bergen, Norway

**Correction: BMC Oral Health 25**,** 1262 (2025)**

**https://doi.org/10.1186/s12903-025-06643-6**

The PDF version of this originally published article [1] was the uncorrected proof; it has now been replaced by the corrected version.

The original article has been corrected.
